# Three-dimensional surface imaging in breast cancer: a new tool for clinical studies?

**DOI:** 10.1186/s13014-020-01499-2

**Published:** 2020-02-28

**Authors:** Konstantin Christoph Koban, Lucas Etzel, Zhouxiao Li, Montserrat Pazos, Stephan Schönecker, Claus Belka, Riccardo Enzo Giunta, Thilo Ludwig Schenck, Stefanie Corradini

**Affiliations:** 1 Division of Hand, Plastic and Aesthetic Surgery, University Hospital, LMU Munich, Pettenkoferstraße 8a, 80336 Munich, Germany; 2 Department of Radiation Oncology, University Hospital, LMU Munich, Munich, Germany

**Keywords:** Breast cancer, Breast-conserving therapy, Radiation therapy, Three-dimensional surface imaging, Breast imaging, Clinical studies, Volume measurements, Skin, Toxicity, Outcome

## Abstract

**Background:**

Three-dimensional Surface Imaging (3DSI) is a well-established method to objectively monitor morphological changes in the female breast in the field of plastic surgery. In contrast, in radiation oncology we are still missing effective tools, which can objectively and reproducibly assess and document adverse events in breast cancer radiotherapy within the framework of clinical studies. The aim of the present study was to apply structured-light technology as a non-invasive and objective approach for the documentation of cosmetic outcome and early effects of breast radiotherapy as a proof of principle.

**Methods:**

Weekly 3DSI images of patients receiving either conventionally fractionated radiation treatment (CF-RT) or hypofractionated radiation treatment (HF-RT) were acquired during the radiotherapy treatment and clinical follow-up. The portable Artec Eva scanner (Artec 3D Inc., Luxembourg) recorded 3D surface images for the analysis of breast volumes and changes in skin appearance. Statistical analysis compared the impact of the two different fractionation regimens and the differences between the treated and the contralateral healthy breast.

**Results:**

Overall, 38 patients and a total of 214 breast imaging sessions were analysed. Patients receiving CF-RT showed a significantly higher frequency of breast erythema compared to HF-RT (93.3% versus 34.8%, *p* = 0.003) during all observed imaging sessions. Moreover, we found a statistically significant (*p* < 0.05) volumetric increase of the treated breast of the entire cohort between baseline (379 ± 196 mL) and follow-up imaging at 3 months (437 ± 224 mL), as well as from week 3 of radiotherapy (391 ± 198 mL) to follow-up imaging. In both subgroups of patients undergoing either CF-RT or HF-RT, there was a statistically significant increase (*p* < 0.05) in breast volumes between baseline and 3 months follow-up. There were no statistically significant skin or volumetric changes of the untreated healthy breasts.

**Conclusions:**

This is the first study utilizing 3D structured-light technology as a non-invasive and objective approach for the documentation of patients receiving breast radiotherapy. 3DSI offers potential as a non-invasive tool to objectively and precisely monitor the female breast in a radiooncological setting, allowing clinicians to objectively distinguish outcomes of different therapy modalities.

## Introduction

Adjuvant radiation therapy (RT) offers a significant benefit in preventing local recurrences and improves cancer-specific survival in patients undergoing breast-conserving therapy for early-stage breast cancer [1, 2]. Over the past decade the evaluation of hypofractionated treatment protocols has been of major interest in modern breast radiotherapy [3]. Several randomized trials (START A, START B trials and the Canadian trial) [4, 5] proved the safety and efficacy of hypofractionation (HF-RT), which recently has been introduced as the new standard of care in clinical practice [4–7]. Historically, the standard regimen consisted of conventional fractionated RT (CF-RT) using 25 fractions up to a dose of 50.0 Gy. Regarding primary endpoints like locoregional tumor relapse rates, there were no significant differences between the fractionation regimens in the randomized trials. However, regarding adverse effects, the HF-RT showed significantly less toxicities (breast induration/shrinkage, telangiectasia, and breast oedema) as compared to CF-RT. [8, 9] Most studies used clinical assessments, standardized questionnaires or standardized photographs to assess cosmetic outcome and adverse effects of breast RT. [10, 11] Nevertheless, it remains difficult to objectively assess side effects, like erythema, edema or fibrosis, which can negatively influence the shape, symmetry and appearance of the breast, and subsequently the patient’s quality of life.

Tools that can objectively assess and document adverse events in order to identify the impact of different treatment regimens are therefore becoming increasingly important for future clinical studies. Recent technological developments have enabled the use of three-dimensional surface imaging (3DSI) as a viable solution to assess changes in breast contour and appearance [12–14]. 3DSI are powerful imaging devices that have already found widespread application in the field of plastic surgery, where they are advantageously affecting the process of planning and documenting breast-surgical procedures. One of the used imaging methods is depth sensor-based tracking of the body contour, which is also used for Surface Image Guided Radiation Therapy [15–18]. Other technologies, such as stereo-photogrammetric or structured light 3DSI, are also viable options for clinical applications [19]. The mobile Artec Eva 3D surface scanner (Artec 3D Inc., Luxembourg) is an affordable device that combines the latter two technologies. Its ability to create both, spatially highly accurate and textured 3D models with little effort, has already been successfully used in other clinical studies [20–23]. 3DSI allows to reproducibly assess changes in breast volume and appearance due to edema, fibrosis or erythema.

The aim of the present study was the application of an established 3DSI-based method routinely used in plastic surgery to document and quantify changes of the breast in patients receiving CF-RT and HF-RT, and to objectively confirm early onset of adverse effects during radiotherapy and early clinical follow-up.

## Patients, materials and methods

### Patient inclusion

Patients were invited to participate in this study on the day of their first visit at the department of Radiation Oncology, University Hospital, LMU Munich, Germany. Written informed consent was obtained from all patients. Patients receiving simultaneous bilateral breast radiotherapy were excluded from participation.

### Radiation treatment

The breast clinical target volume (CTV) was delineated encompassing the glandular tissue of the breast, while the planning target volume (PTV) was defined by adding a margin of 5 mm in the transverse plane and 8 mm in cranial–caudal direction to the CT, according to the European Society for Radiotherapy and Oncology (ESTRO)-guidelines [24]. 3-dimensional conformal radiation therapy (3D-CRT) treatment planning was performed using the Oncentra Masterplan treatment planning system version 4.5.2 (Elekta AB, Sweden). All plans consisted of two opposing tangential beams for the breast with the addition of some subfields to increase dose homogeneity or to add the dose for the simultaneous integrated boost (SIB). The conventional fractionated RT (CF-RT) consisted of a total dose of 50 Gy in 25 fractions, while the hypofractionated regimens (HF-RT) was applied up to a dose of 40.05 Gy in 15 fractions. The boost to the tumor bed consisted of 10–16 Gy in 2 Gy single dose or a simultaneously integrated boost with a single dose of 0.5Gy/daily.

### Imaging devices

**Fig. 1:**
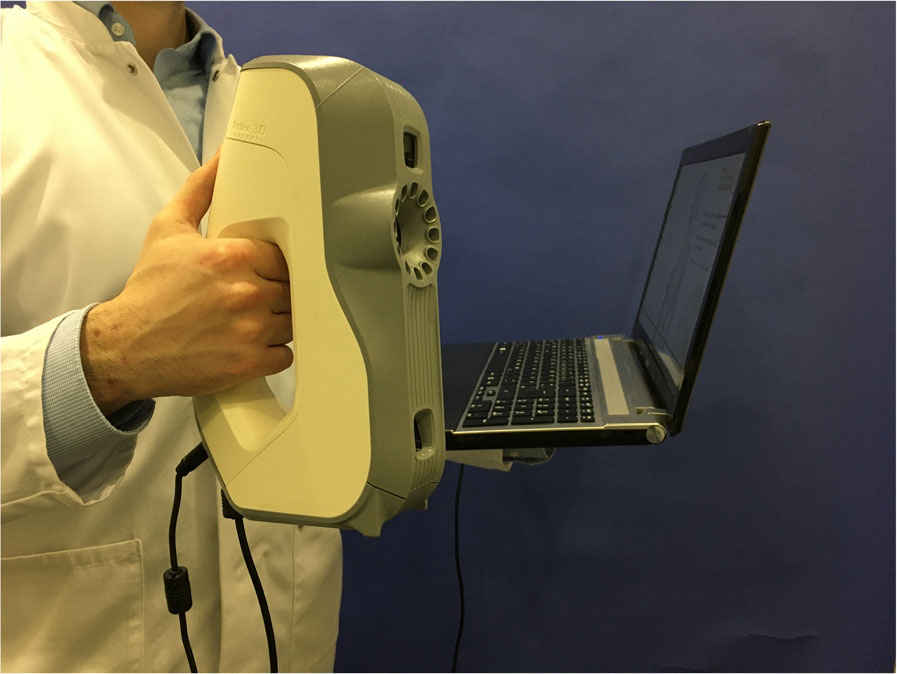
The mobile structured-light surface scanner Artec Eva (Artec 3D Inc., Luxembourg) used for all imaging procedures

The portable Artec Eva capture device (Artec 3D Inc., Luxembourg) was used for non-invasive 3DSI. It is depicted in Fig. 1. The surface scanner is equipped with structured light technology, which allows for a 3D point accuracy of up to 0.1 mm and a 3D resolution of up to 0.5 mm as specified by the manufacturer. With an operating distance ranging between 0.4 and 1 m, it enables the operator to rapidly capture localized areas of the human body [21–23, 25–27]. For the duration of the imaging process, the surface scanner was tethered via USB-interface to a commercially available capture Dell XPS 159560 laptop. The compact device was equipped with the Artec Studio Professional 12 software (Artec 3D Inc., Luxembourg) for surface capture and enabled subsequent image processing and analysis.

### 3D imaging and processing

One of three skilled operators (KCK, LE or ZL) informed the patients about the imaging procedure before conducting the baseline imaging session prior to the first radiation treatment. Subsequent images were acquired in weekly intervals for the duration of the treatment, as well as during the routine clinical follow-up where applicable.

**Fig. 2:**
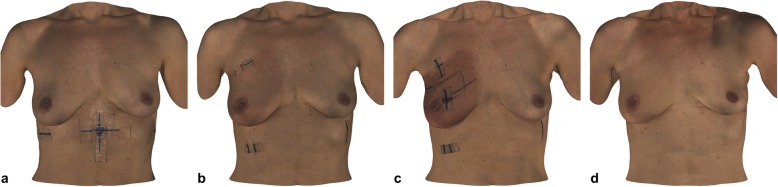
Three-dimensional surface imaging overview of the front-facing torso of a patient receiving 50 Gy conventional fractionated radiotherapy with a subsequent 16 Gy boost of the right breast, as can be seen during baseline (a), week 3 (b), week 5 (c) and 3 months follow-up (d) imaging

**Fig. 3:**
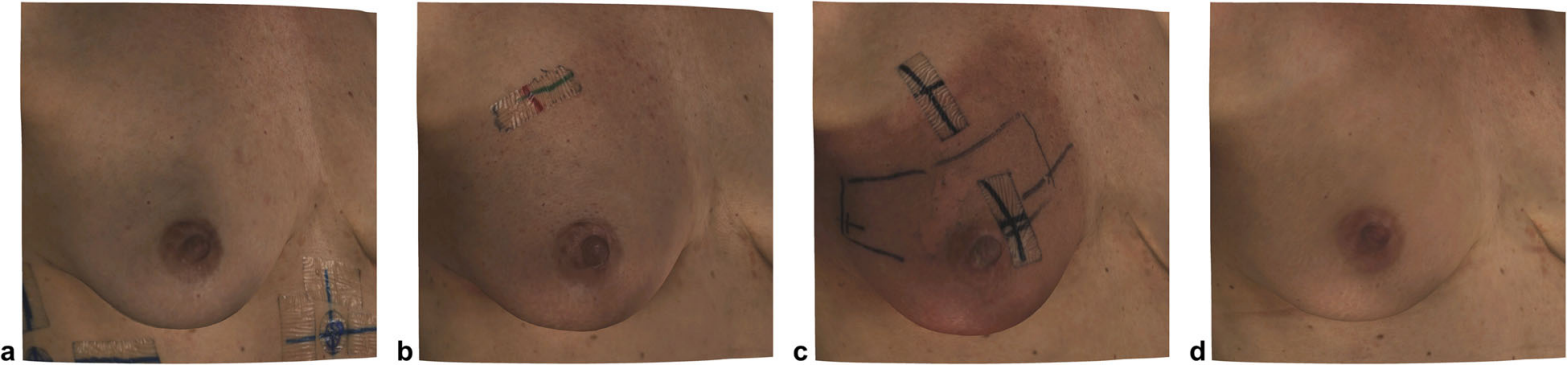
Close-up image of the irradiated breast of the patient shown in Fig. 2, depicting in detail the breast appearance during baseline (a), week 3 (b), week 5 (maximum intensity of erythema) (c) and 3 months follow-up (d) imaging

Following a standardized protocol for patient positioning, imaging of the breast was conducted in an upright standing position. The patient’s hands were placed on the hips, while the arms were left relaxed and the face was directed straight ahead. To prevent surface artefacts, the patients were requested to remove necklaces and clothing covering the torso prior to surface imaging. The operator instructed the patients to remain in the designated position and to keep as still as possible while breathing freely. Holding the surface scanner in one hand and the laptop placed on a side table directly adjacent to the patient, the operator proceeded to systematically capture all visible areas of the breast and front-facing torso. Imaging duration averaged to about 30 s. The raw data file was saved upon scan conclusion, followed by later registration and correct alignment of individual frames of the chest image resulting in a reconstruction of a textured 3D data file. An example of the final surface images can be seen in Figs. 2 and 3.

### Assessment of breast changes

Usually, early toxicity and breast changes already occur during fractionated RT or within the first 12 weeks following RT. Skin erythema is the first sign of radiation dermatitis and its intensity varies with the radiation dose. While transient erythema may be seen even after a single fraction of radiation (2 Gy), hyperpigmentation, epitheliolysis, and desquamation only occur with increasing RT dose [28].

Late changes are considered to occur after 12 weeks following RT. There may be a variable latency period following acute/early changes during which the skin may appear normal and late effects like xeroderma, atrophy, telangiectasia, subcutaneous fibrosis which may develop after years. Fibrosis may develop, with progressive induration, edema, and thickening of the dermis and subcutaneous tissues [29]. The total radiation dose is critical in determining the severity of acute skin reactions, while the late effects are more influenced by the dose per fraction. Possible changes of the breast appearance as stated above were observed and recorded by the analysis of the textured 3D images after the conclusion of data acquisition.

### 3D volumetric analysis

The digital surface images of each patient were exported in the OBJ file format and superimposed by use of the closest iterative point algorithm within the Mirror Medical Imaging software (Canfield Sci., NJ, USA). Digital landmarks were placed by a trained examiner (KCK) for further analysis of breast changes. Breast dimensions were acquired by an automated software log reporting on the distances over surface between landmarks. Individual breast volume was quantified using another integrated software algorithm that digitally interpolated the chest wall to calculate the respective volume.

### Statistical analysis

We examined the breast appearance as well as volumetric breast measurements of all irradiated breasts. Changes during RT treatment and follow-up were analysed for all patients, as well as for different subgroups. The baseline was statistically compared with the subsequent imaging sessions and the clinical follow-up imaging. The untreated contralateral breasts were volumetrically analysed as control measurements to confirm scanner reproducibility. Subgroup analysis was performed regarding fractionation regimen (CF-RT vs HF-RT).

The frequency of changes in breast appearance was assessed using Fisher’s exact test methodology. Changes regarding breast volume were examined using one-way repeated measures ANOVA for data where the assumptions of normal distribution and equal variance were met, else the Friedman repeated measures ANOVA on ranks was used. A *p*-value < 0.05 was considered statistically significant. All statistical analyses were conducted using the SigmaPlot Version 12.0 software package for scientific graphing and data analysis (Systat Software Inc., San José, CA, USA).

## Results

### Patient and tumor characteristics

For this study we enrolled thirty-eight (38) female patients. All women underwent radiation treatment and were imaged using 3DSI between November 2017 and September 2018. Patient and treatment characteristics are summarized in Table 1. Median age at diagnosis was 57 years (range: 30–80) and the median body mass index (BMI) was 23.3 (range: 17.2–28.5). The tumor side was right-sided in 16 cases (42.1%) and left-sided in 22 patients (57.9%). Overall, tumor stage was pTis in 4/38 women (10.6%), pT1 in 22/38 patients (57.9%), pT2 in 4/38 cases (10.6%) and pT3 in 1/38 patients (2.6%). Nodal status was negative in the majority of patients (78.9%, 30/38), positive in 5 patients (13.2%) and not evaluated in 3 patients (7.9%) with the diagnosis of DCIS (*n* = 2) or in the setting of recurrent disease (*n* = 1). Eleven patients underwent neoadjuvant systemic therapy due to triple negative breast cancer (*n* = 4), Her2-neu positive disease (*n* = 6) or a locally advanced cT4b tumor (n = 1).

**Table 1:** Cohort characteristics

		38 patients	
		n	(%)
Age at diagnosis (years)	< 50	9	(23.7)
	51–60	13	(34.2)
	61–70	6	(15.8)
	> 71	10	(26.3)
	median age (years)	57.0	
Tumor side	Left	22	(57.9)
	Right	16	(42.1)
Surgery	BCS	36	(94.7)
	Mastectomy with immediate reconstruction	2	(5.3)
Tumour size	ypT0	7	(18.4)
	pTis	4	(10.6)
	pT1	22	(57.9)
	pT2	4	(10.6)
	pT3	1	(2.6)
Nodal status	pN0	30	(78.9)
	pN+	5	(13.2)
	pNx	3	(7.9)
Grade	G1	5	(13.2)
	G2–3	33	(86.8)
Hormone Receptor	positive	27	(71.1)
	negative	10	(26.3)
	unknown (DCIS)	1	(2.6)
Her2/neu Status	positive	9	(23.7)
	negative	27	(71.0)
	unknown (DCIS)	2	(5.3)
Radiotherapy regimen	Normofractionated (50Gy/25fx)	15	(39.5)
	Hypofractionated (40Gy/15fx)	23	(60.5)
Boost	no	21	(55.3)
	yes	17	(44.7)
BMI	median (range)	22.3	(17.2–28.5)

Regarding the fractionation schemes, 15/38 patients (38.5%) received a normofractionated RT regimen (50Gy/25fractions) and 23/38 patients (60.5%) a hypofractionated RT (40Gy/15fx). Moreover, 17 patients (44.7%) received a consecutive boost irradiation to the tumor bed or a simultaneously integrated boost (n = 2).

### Assessment of breast changes

A total of 214 imaging sessions were conducted. None of the patients receiving CF-RT or HF-RT showed signs of a skin reaction during the baseline imaging session. Details on the incidence of changes in breast appearance are displayed in Table 2.

**Table 2:** Assessment of breast changes (skin erythema) during the treatment course and clinical follow-up at 3 months

*Timepoint*	*baseline*	*week 1*	*week 2*	*week 3*	*week 4*	*week 5*	*week 6*	*week 7*	*follow-up*
*Overall*	*0.0*	*2.9*	*14.7*	*36.8*	*77.3*	*100.0*	*100.0*	*66.7*	*3.7*
CF*-RT*	*0.0*	*0.0*	*7.7*	*46.7*	*92.9*	*100.0*	*100.0*	*66.7*	*0.0*
*HF-RT*	*0.0*	*4.8*	*19.0*	*30.4*	*50.0*	*NA*	*NA*	*NA*	*5.6*

All results are expressed in percent of patients (*n* = 38)

When examining the overall changes regarding breast appearance, we found localised skin erythema in 22 out of 38 cases (57.9%). One patient (2.6%) of the entire cohort suffered from epitheliolysis of the nipple-areolar complex, which subsided completely before the final follow-up imaging session at 3 months after RT. Moreover, all skin reactions were observed during a maximum of 4 consecutive imaging sessions. Changes in breast appearance were mostly self-limited after the end of the radiation treatment with only one observed case (3.7%) of persistent erythema during clinical follow-up.

Regarding the influence of the different fractionation schemes, 1 out of 15 patients receiving CF-RT (6.7%) showed no signs of skin reaction, while 14 out of 15 women (93.3%) experienced skin erythema within the target volume (see Figs. 2 and 3). The median starting point was during the third week of radiation, with a maximum peak during the fifth week. The erythema decreased until the sixth week of radiation treatment and had a median duration of 2 weeks. In contrast, 15 out of 23 patients undergoing HF-RT (65.2%) showed no signs of any skin reaction, while eight out of 23 patients (34.8%) exhibited localised skin erythema. The median starting point was earlier than in CF-RT and began in the second week of radiation, with a maximum peak in the third week and the end point during the fourth week of radiation treatment. The duration of the skin reaction was similar to CF-RT, with a median duration of 2 weeks.

We observed changes in breast appearance during week 3 of RT in 46.7% of patients receiving CF-RT, and in 30.4% of patients receiving HF-RT. When examined using Fisher’s exact test methodology, this difference was not statistically significant (*p* = 0.165). None of the patients undergoing CF-RT presented with changes in breast appearance during the clinical follow-up visit, whereas one single HF-RT patient (5.6%) exhibited persistent localised redness. This difference was also not statistically significant (*p* = 0.805). However, patients receiving CF-RT showed a statistically significant higher frequency of changes in breast appearances when compared to HF-RT (93.3% versus 34.8%, *p* = 0.003) if all observed imaging sessions were considered.

### 3D volumetric analysis

The detailed volumetric results are listed in Table 3. The mean breast volume for the entire cohort during baseline imaging was 379 ± 196 mL, as compared to 391 ± 198 mL during week 3 of RT and 437 ± 224 mL during clinical follow-up at 3 months. When using the Friedman test to compare the volumetric results of the treated breast for the entire cohort, we found a statistically significant change in breast volume between the examined imaging sessions (*p* < 0.001). Subsequent use of Dunn’s method of multiple comparisons showed a statistically significant increase (*p* < 0.05) of breast volumes between both, baseline and follow-up at 3 months, as well as between week 3 and follow-up imaging. There were no statistically significant changes in breast volume when examining the untreated healthy breasts (*p* = 0.708).

**Table 3:** Assessment of volumetric breast changes during the treatment course and clinical follow-up at 3 months for the entire cohort and the two subgroups, receiving either conventionally fractionated RT (CF-RT) or hypofractionated RT (HF-RT)

	*baseline*	*week 3*	*follow-up*
*Entire cohort (treated breast)*	*379 ± 196*	*391 ± 198*	*437 ± 224*
*Entire cohort (untreated breast)*	*382 ± 203*	*382 ± 205*	*382 ± 208*
CF*-RT (treated breast)*	*387 ± 171*	*416 ± 183*	*443 ± 212*
CF*-RT (untreated breast)*	*363 ± 174*	*370 ± 184*	*338 ± 160*
*HF-RT (treated breast)*	*375 ± 211*	*379 ± 209*	*434 ± 236*
*HF-RT (untreated breast)*	*392 ± 220*	*389 ± 220*	*404 ± 229*

All results are given as mean ± sd (standard deviation) in millilitres (mL)

Regarding the influence of the different fractionation schemes, in patients undergoing CF-RT, the mean breast volume during baseline imaging was 387 ± 171 mL, as compared to 416 ± 183 mL during week 3 and 443 ± 212 mL during clinical follow-up at 3 months (p < 0.001). Subsequent analyses showed a statistically significant increase (p < 0.05) of breast volumes between baseline and follow-up imaging. Similarly, patients undergoing HF-RT, had a mean breast volume during baseline imaging of 375 ± 211 mL, 379 ± 209 mL during week 3, and 434 ± 236 mL during clinical follow-up at 3 months, which was a statistically significant increase (p < 0.05) between baseline and follow-up imaging.

## Discussion

In the present study, weekly 3DSI of patients undergoing either CF-TR or HF-RT was conducted for the duration of RT treatment and on clinical follow-up. The portable Artec Eva scanner provided reproducible 3D surface images for analysis of breast volume and changes in breast appearance. A total of 38 patients were enrolled and 214 breast imaging sessions were performed.

While a greater percentage of patients receiving CF-RT showed changes in breast appearance during week 3 of RT when compared to patients receiving HF-RT (46.7% versus 30.4%), this difference was not statistically significant (p = 0.165). No patients having received CF-RT presented with changes in breast appearance during the clinical follow-up visit, whereas a single patient having received HF-RT, exhibited localised redness (0.0% versus 5.6%, p = 0.805). However, patients receiving CF-RT showed a statistically significant higher frequency of changes in breast appearances when compared with HF-RT (93.3% versus 34.8%, p = 0.003) throughout all observed imaging sessions.

When assessing the volumetric breast changes during the treatment course and clinical follow-up at 3 months for the entire cohort, we found a statistically significant (p < 0.05) volumetric increase between both baseline and follow-up imaging, as well as between week 3 and follow-up imaging. In both subgroups of patients undergoing either CF-RT or HF-RT, there was a statistically significant increase (p < 0.05) in breast volumes between baseline and 3 months follow-up imaging. As a proof of principle, we found no statistically significant changes in breast volumes when examining the untreated healthy breast.

To the best of our knowledge, this is the first study utilizing 3D structured-light technology as a non-invasive and objective approach for the documentation of side effects during breast radiotherapy. Quantitative metrics derived from 3D surface images allowed to compare the differences regarding cosmetic outcome and early effects of radiation toxicity of different fractionation regimens. While the use of 3DSI enables the assessment of the occurrence and development of skin changes, such as hyperpigmentation and erythema, the software lacks an automated algorithm to analyse the chromatic properties of these changes. Although such tools have been described in other works [30, 31], the application of such tools in conjunction with standardized 3DSI might further improve objective assessments in future. Applying this technology in clinical studies in the field of radiation oncology could offer an additional, standardized method to objectively assess and document side effects, such as variations in breast volume or skin deterioration, and match those to subjective evaluated parameters of patient reported outcome measurements (PROMS).

In comparison to conventional photography, studies have shown the superiority of objective and reproducible measurements of 3DSI [32, 33]. The 3D imaging device used in the present setting is a portable system that works with structured light imaging and photogrammetric technology, thus enabling fast capturing of a complete 3D surface model of the front-facing thorax within 30 s. During the scanning process itself, the distance from the patient to the camera is shown on the computer screen for better reproducibility. In addition, the practicability is very good, as data processing takes only 2–5 min until final evaluation. This could be even faster, if a more powerful computer was used in clinical practice. However, a drawback of the Artec software is the lack of an accompanying user-friendly medical patient database and comprehensive assessment and evaluation tools for detailed analysis. By importing the scans into the Mirror medical imaging software, we were able to use its database function and analysis tools for greater ease of use.

The texture quality is excellent even under normal light conditions and allows a detailed assessment of skin structure. The system has an integrated light source, which provides a high frequency sequential flashing light, which eliminates the need for extra light adjustments in the background. However, the sequential flashing lights could be unpleasant during patient consultation and its use is not recommended in patients suffering from epilepsy. Regarding susceptibility to involuntary movements, sensitivity was significantly reduced when patients were asked to remain in a relaxed body pose.

The volumetric measurement capabilities of the Artec Eva scanner were previously examined in a cadaveric model, where predefined amounts of injected volume were analysed in various facial regions [20, 21]. The imaging device was found to provide highly accurate results, even when dealing with as little as milliltres of injected volume. Thus, it can be assumed that the scanner used in this study provides very reliable results regarding volumetric changes of both the irradiated as well as the untreated breast.

Nevertheless, the system has also some remaining limitations. Although 3DSI offers a low-priced and radiation-free method of assessing body contour and volumetric developments when compared to MRI or CT, the technology only allows body surfaces to be registered. For this reason, it is necessary to compare at least two images to document the relative difference between surfaces. Depending on the technology and software used for imaging, measurements regarding absolute breast volumes have been known to show a higher variance when compared to MRI and CT, and therefore remain inconclusive [34]. Another limitation is the process of manual landmark placement to digitally mark breast boundaries in patients with a high BMI or severe breast ptosis, especially when regarding the inframammary fold and lateral breast border. In the present study, the breast volume was measured by digitally calculating the space enclosed by the 3D surface image of the breast and a digital surface representing the chest wall. The spatial accuracy of this interpolated surface relies heavily on correctly identified breast borders. However, a trained examiner performed the placement of landmark points using a standardized operating procedure, and as a method for internal validation and proof of principle, the volume of untreated contralateral breasts was measured to confirm reproducibility. Over the entire study duration, there were no significant volume changes in the respective healthy contralateral breasts. Physiological volumetric changes of the healthy breast could occur due to changes in body weight or through the influence of the menstrual cycle [35], although they were observed to be negligibly small. Thus, the surface scanner can be assumed to precisely gauge the captured breast volume.

## Conclusion

This is the first study utilizing 3D structured-light technology as a non-invasive and objective approach for the documentation of patients receiving breast radiotherapy. Quantitative metrics derived from 3D surface images allowed to compare the differences regarding cosmetic outcome and early effects of conventionally fractionated (CF-RT) and hypofractionated radiation treatment (HF-RT). 3DSI offers potential as a non-invasive tool to objectively monitor the female breast in a radiooncological setting, allowing clinicians to objectively and precisely distinguish outcomes of different therapy modalities.

## Data Availability

The datasets generated and/or analysed during the current study are not publicly available due to the General Data Protection Regulation (GDPR) but are available from the corresponding author on reasonable request.
